# Epigenetic regulation of SMAD3 by histone methyltransferase SMYD2 promotes lung cancer metastasis

**DOI:** 10.1038/s12276-023-00987-1

**Published:** 2023-05-01

**Authors:** Kwangho Kim, Tae Young Ryu, Eunsun Jung, Tae-Su Han, Jinkwon Lee, Seon-Kyu Kim, Yu Na Roh, Moo-Seung Lee, Cho-Rok Jung, Jung Hwa Lim, Ryuji Hamamoto, Hye Won Lee, Keun Hur, Mi-Young Son, Dae-Soo Kim, Hyun-Soo Cho

**Affiliations:** 1 Korea Research Institute of Bioscience and Biotechnology, Daejeon, Republic of Korea; 2 College of Pharmacy, Chungnam National University, Daejeon, Republic of Korea; 3 Department of Functional Genomics, Korea University of Science and Technology, Daejeon, Republic of Korea; 4 Department of Biological Science, Sungkyunkwan University, Suwon, Republic of Korea; 5 Division of Molecular Modification and Cancer Biology, National Cancer Center, Tokyo, Japan; 6 Department of Pathology, Keimyung University School of Medicine, Daegu, Republic of Korea; 7 Department of Biochemistry and Cell Biology, School of Medicine, Kyungpook National University, Daegu, Republic of Korea

**Keywords:** Metastasis, Gene silencing, Lung cancer

## Abstract

Epigenetic alterations, especially histone methylation, are key factors in cell migration and invasion in cancer metastasis. However, in lung cancer metastasis, the mechanism by which histone methylation regulates metastasis has not been fully elucidated. Here, we found that the histone methyltransferase SMYD2 is overexpressed in lung cancer and that knockdown of SMYD2 could reduce the rates of cell migration and invasion in lung cancer cell lines via direct downregulation of SMAD3 via SMYD2-mediated epigenetic regulation. Furthermore, using an in vitro epithelial-mesenchymal transition (EMT) system with a Transwell system, we generated highly invasive H1299 (In-H1299) cell lines and observed the suppression of metastatic features by SMYD2 knockdown. Finally, two types of in vivo studies revealed that the formation of metastatic tumors by shSMYD2 was significantly suppressed. Thus, we suggest that SMYD2 is a potential metastasis regulator and that the development of SMYD2-specific inhibitors may help to increase the efficacy of lung cancer treatment.

## Lung cancer: An opportunity to prevent cancer spread

A protein that accelerates cancer spread (metastasis) could be an effective target for novel therapies for non-small-cell lung cancer (NSCLC), which generally has a poor prognosis. Increased activity of the SMYD2 protein is associated with more aggressive growth in several cancer types, and Kwangho Kim of the Korea Research Institute of Bioscience and Biotechnology, Daejeon, and colleagues have linked heightened SMYD2 expression with poor prognosis in NSCLC patients. Experiments with cultured lung cancer cells demonstrated that SMYD2 plays a role in epithelial-mesenchymal transition, a cellular transformation process that leads to metastatic growth. Interventions that disrupt SMYD2 production in tumor cells hindered this process. Mice transplanted with SMYD2-deficient cells were significantly less prone to metastases than animals receiving cells with unimpeded SMYD2 production. These findings could present a therapeutic approach for this form of lung cancer.

## Introduction

Lung cancer is the most common type of cancer worldwide, and non-small cell lung cancer (NSCLC), which includes adenocarcinoma and squamous cell carcinoma, accounts for approximately 80% of lung cancer cases. NSCLC has a poor prognosis and a very low 5-year survival rate because of its highly malignant characteristics and nonspecific symptoms. Cisplatin, paclitaxel, and gemcitabine are used for chemotherapy in NSCLC. However, severe side effects and lung cancer recurrence are becoming serious problems for lung cancer treatment. Therefore, to improve NSCLC treatment, the development of new therapeutic targets and diagnostic markers is still required^1,2^.

In cancer progression and metastasis, epigenetic regulation, specifically histone methylation, is known to be a critical aspect in the control of gene expression. The opening of the chromatin structure by activating histone methylation (methylation of histone H3 lysines 4 and 36) and the closing of the chromatin structure by repressive histone methylation (methylation of histone H3 lysines 9 and 27) are continuously changed in response to regulation by oncogenes and tumor suppressive genes^3^. Recently, the FDA approved tazemetostat, a specific inhibitor of enhancer of zeste 2 polycomb repressive complex 2 subunit (EZH2) activity, for follicular lymphoma and epithelioid treatment^4^, and various histone methyltransferases have been studied as therapeutic targets for cancer treatment^4–8^. Among them, SET and MYND domain containing 2 (SMYD2) is a histone methyltransferase involving the MYND and SET domains, which induce conformational changes in the euchromatin structure to upregulate target genes via methylation of H3K36 (gene body region) and H3K4 (promoter region)^9^. In lung cancer, downregulation of SMYD2 induces suppression of cell growth in cisplatin-resistant lung cancer cells^10^. Additionally, SMYD2 overexpression induces glycolytic metabolism in cervical cancer^11^ and is critically related to cell apoptosis, and this results is also supported by knockdown experiments in colon and ovarian cancers^12–14^. In addition, SMYD2 overexpression accelerates gastrointestinal stromal tumors through upregulation of EZH2 and downregulation of TET1^15^. In the metastasis process, SMYD2 induces the epithelial-mesenchymal transition (EMT) process via activation of the Wnt/β-catenin pathway in colon cancer^16^. Moreover, in cancer-related nonhistone methylation, SMYD2 methylates the RB1, ALK, and β-catenin proteins to regulate the cell cycle and cell proliferation, implying that SMYD2 is a potential therapeutic target for cancer treatment^17–20^. Although many reports have suggested that SMYD2 is related to cancer progression, metastasis regulation by SMYD2, especially in lung cancer, is not fully understood.

SMAD family member 3 (SMAD3) is a key molecule in the regulation of cancer metastasis-related genes, such as ZEB, Twist family genes, and Snail, via the formation of the TGF-β-induced SMAD 2/3/4 complex in several types of cancer^21^. In lung cancer, profiling-2 and ACP5 regulate Smad2 and Smad3 expression for lung cancer growth and metastasis^22,23^, and miR-15a and miR-32-5p directly regulate SMAD3 expression for lung cancer metastasis^24,25^. Moreover, the long noncoding RNA HCP5 induced by SMAD3 regulates lung adenocarcinoma metastasis by sponging miR-203, implying that SMAD3 is a main target molecule for the inhibition of lung cancer metastasis.

Thus, in this study, we hypothesized that SMYD2 was overexpressed in lung cancer and identified SMAD3 as a direct target of SMYD2 via epigenetic regulation that promotes lung cancer metastasis. In particular, an in vitro EMT system was used to generate highly invasive lung cancer cell lines, and the results showed that SMYD2 knockdown clearly suppressed migration and invasion. Furthermore, in two in vivo metastasis studies, downregulation of SMYD2 inhibited the metastasis of lung cancer cell lines, implying that SMYD2 is a potential regulator of lung cancer metastasis.

## Materials and methods

### Cell Culture and Reagents

The human lung cancer cell lines H1299 and H1703 were purchased from the Korean Cell Line Bank (Seoul, South Korea) and cultured in RPMI supplemented with 10% fetal bovine serum (FBS) and 1% penicillin/streptomycin in a humidified atmosphere with 5% CO_2_ at 37 °C. LLY507 (HY-19313) was purchased from MedChemExpress. To establish luciferase-expressing H1299 cells (shCont and shSMYD2), H1299 cells were infected with F-luc lentivirus (Capital Biosciences, VSL-0044), and the cells were selected with puromycin (2 μg/ml) for 2 weeks.

### Selection of invasive cells

A 6-well transparent PET membrane with an 8.0-µM pore size (Corning, #353093) was used for the selection of migrated H1299 cells. Six-well inserts were coated with 1.5 ml of 2% gelatin (Biosolution, #BG008-1). A total of 8 × 10^5^ H1299 cells were seeded into the inner well, and 2.5 ml of RPMI 1640 medium supplemented with 20% FBS was added into the outer well. After incubation for 48 h at 37 °C, the migrated cells on the outer membrane were harvested using trypsin EDTA, and the above process was repeated for ten rounds of selection.

### siRNA transfection

siRNA duplexes against SMYD2 (siSMYD2#1; 5′-GAUUUGAUUCAGAGUGACATT-3′, 5′-UGUCACUCUGAAUCAAAUCTT-3′) (siSMYD2#2; 5′-GUCACUCCAGCAUCUCUG UTT-3′, 5′-ACAGAGAUGCUGGAGUGACTT-3′) were purchased from Bioneer Co., Ltd. (Daejeon, South Korea). siRNA duplexes against SMAD3 (siSMAD3; 5′-CUCAGACCUGAAGGCUACUTT −3′, 5′-AGUAGCCUUCAGGUCUGAGTT −3′) were purchased from Bioneer Co., Ltd. (Daejeon, South Korea). Negative control siRNA (siCont; 5′-AUGAACGUGAAUUGCUCAATT-3′, 5′-UUGAGCAAUUCACGUUCAUTT-3′) was used as a control treatment. The siRNAs (100 nM) were transfected into cancer cell lines using RNAiMax (Invitrogen, Carlsbad, CA) for 48 h.

### Stable expression of shRNA for SMYD2

The two small interfering RNAs (21 nucleotides) were designed against human SMYD2 messenger RNA (mRNA), and the SMYD2 targeting sequences were as follows: siCont, 5′-AUGAACGUGAAUUGCUCAATT-3′; SMYD2#1, 5′-GAUUUGAUUCAGAGUGACAT T-3′. Target sequences were cloned into the pLL3.7 lentiviral vector (graciously provided by Luk Van Parijs, Department of Biology, MIT). For the production of lentiviruses, HEK293FT cells were transfected with pLL3.7 plasmids containing shSMYD2 or shCont (scramble) sequences using Lipofectamine 2000 and OptiMEM media (Invitrogen, Carlsbad, CA, USA) according to the manufacturer’s instructions. After 24–48 h of transfection, the medium was collected from HEK293FT cells, and then the medium was used to infect H1229-luc cells. The filtered viral supernatant was added to 1 × 10^6^ H1299-luc cells in a 10-cm dish, and then transduced cells were selected with blasticidin (30 μg/ml) to obtain shSMYD2-expressing cells.

### Quantitative real-time PCR

Total RNA was isolated from the indicated cell lines using a Qiagen RNeasy Mini Kit according to the manufacturer’s instructions. RNA aliquots of 1 µg were then reverse transcribed using the iScript™ cDNA synthesis kit (Bio-Rad Laboratories, Inc., Hercules, CA, USA) according to standard protocols. Quantitative real-time PCR was performed on cDNA samples using Brilliant III Ultra-Fast SYBR® Green QPCR Master Mix (Agilent Technologies), and the signal was detected with an AriaMx Real-time PCR System (Agilent Technologies). The fluorescence threshold value was calculated using Agilent Aria 1.6 software. The PCR primers used were as follows: SMYD2 (forward, 5′-ATCTCCTGTACCCAACGGAAG-3′ and reverse, 5′-CACCTTGGCCTTATCCTTGTCC-3′), SMAD3 (forward, 5′-TGGACGCAGGTTCTCCAAAC-3′ and reverse, 5′-CCGGCTCGCAGTAGGTAAC-3′), CDH2 (forward, 5′-AGCCAACCTTAACTGAGGA GT-3′ and reverse, 5′-GGCAAGTTGATTGGAGGGATG-3′), Vimentin (forward, 5′-CCCTCACCTGTG AAGTGGAT-3′ and reverse, 5′-TGACGAGCCATTTCCTCCTT-3′), Claudin1 (forward, 5′-TGGTCAGGCTCTCTTCACTG-3′ and reverse, 5′-TTGGATAGGGCCTTGGTGTT-3′), MMP-9 (forward, 5′-TCCAGTACCGAGAAAGCC-3′ and reverse, 5′-CATAGGTCACGTAGCCCACT-3′), ACTB (forward, 5′-ACTCTTCCAGCCT TCCTTCC-3′ and reverse, 5′-CAATGCCAGGGTACATGGTG-3′), SMAD2 (forward, 5′-CGTCCATCTTGCCATTCACG-3′ and reverse, 5′-CTCAAGCTCATCTAATCGTCCTG-3′), and SMAD4 (forward, 5′-CTCATGTGATCTATGCCCGTC-3′ and reverse, 5′-AGGTGATACAACTCGTTCGTAGT-3′).

### Migration and invasion assays

Transwell inserts were coated with a 2% gelatin solution and incubated at room temperature for 4 h for the migration assay. The gelatin-coated Transwell inserts (353097, BD Falcon, Bedford, MA) and invasion chambers (354480, Corning, Corning, NY) were rehydrated in serum-free medium. Complete medium with 20% FBS (700 µl) served as a chemoattractant in the bottom chamber. Approximately 1 × 10^5^ cells/well were incubated in the plates for 36 h at 37 °C with 5% CO_2_. At the end of the incubation period, the migrated and invaded cells were fixed with methanol for 5 min and stained with 0.1% crystal violet.

### Wound healing assay

Cells were seeded in 6-well plates and wounded by scratching with sterile plastic 10 µl micropipette tips after 24 h of siRNA infection or inhibitor treatment. Then, the cells were washed with PBS, and fresh serum medium or inhibitor-treated medium was added. The cells were photographed at 0 h, 24 h, and 48 h after wounding by means of the CELENA^TM^ S Digital Imaging System (Logos Biosystems). The cell migration distance was observed in the photographs.

### Western blot analysis

The cells were washed once with PBS and then lysed in cold lysis buffer (50 mM Tris-HCl, pH 7.4, 150 mM NaCl, 1% Triton X-100, 0.1% SDS, 1 mM EDTA, 1 mM Na3VO4, 1 mM NaF and 1× protease inhibitor cocktail). Cell lysates were centrifuged at 14,000 × *g* for 15 min at 4 °C and then boiled in 5× sample buffer following protein determination (BSA, #23208; Thermo Fisher Scientific, Inc.). The protein samples were subjected to western blot analysis. For western blot analysis, nitrocellulose membranes (#1620145; Bio-Rad Laboratories, Inc.), blocking reagent (5% skim milk, 1 h, room temperature), precast gels (#456-1095; Bio-Rad Laboratories, Inc.) and the indicated antibodies at a 1:1000 dilution ratio were used. The samples were stained with rabbit anti-SMYD2 antibody (#9734; Cell Signaling Technology), rabbit anti-SMAD3 antibody (ab40854; Abcam) and mouse anti-ACTB antibody (SC-47778; Santa Cruz) at 4 °C (overnight). Secondary antibodies (rabbit; SC-2357, mouse; SC-516102, Santa Cruz) were incubated at room temperature for 1 h, and ECL solution (#170-5060; Bio-Rad Laboratories, Inc.) was used for visualization.

### Immunocytochemistry

Cultured cells were washed three times with ice-cold PBS and then fixed in 4% paraformaldehyde at room temperature for 10 min. After that, the cells were washed three times with ice-cold PBS, permeabilized in 0.1% Triton X-100 (Sigma–Aldrich) in PBS for 10 min and washed three times with ice-cold PBS. The cells were blocked with 5% bovine serum albumin in PBS for 30 min. Fixed cells were incubated with anti-SMAD3 antibody (ab40854; Abcam) overnight at 4 °C and stained with Alexa Fluor-conjugated secondary antibodies (Life Technologies). Fluorescence images were obtained using a CELENA® S Digital Imaging System (Logos Biosystems).

### Immunohistochemistry

Paraffin-embedded sections of lung tumor tissue array (T8235732–5, BioChain) were processed in a microwave (90 °C) with antigen-retrieval solution (pH 9) (S2367; Dako), treated with a peroxidase-blocking reagent, and then treated with a protein-blocking reagent (K130, X0909; Dako). Tissue sections were incubated with rabbit anti-SMYD2 antibody (#9734 S; CST) followed by incubation with an HRP-conjugated secondary antibody (Dako). Immunoreactivity was visualized with a chromogenic substrate (Liquid DAB Chromogen; Dako). Finally, tissue specimens were stained with Mayer’s hematoxylin solution (Hematoxylin QS; Vector Laboratories) for 5 s to discriminate the nucleus from the cytoplasm. After the mice were sacrificed, the tumors and organs were collected and fixed in 10% formalin for 24 h. Then, the fixed tissues were sectioned and embedded in paraffin. Tissue Section (4 μm) were deparaffinized and then stained with hematoxylin and eosin (H&E), and Ki67 immunochemistry was performed according to a standard protocol. Images of the whole cross section were captured using an EasyScan slide scanner (Motic). Images were analyzed using Motic ImagePlus software (Motic).

### Chromatin immunoprecipitation

ChIP was performed with a Simple ChIP® Plus Sonication Chromatin IP Kit (#56383; CST) following the manufacturers’ instructions. H1299 cells transfected with siCont and siSMYD2 for 48 h were crosslinked with 1% formaldehyde (Sigma–Aldrich) for 10 min at room temperature and quenched with 1× glycine for 5 min at room temperature. Then, the cells were washed with cold 1× PBS (containing 1× Protease Inhibitor Cocktail) and lysed in 1× cell lysis buffer (containing 1× Protease Inhibitor Cocktail). Then, after nuclear extraction, the chromatin solution was sonicated using a Bioruptor® Pico sonication device (B01060010; Diagenode) with 20 cycles of 30 s ON and 30 s OFF to obtain 200–1000-bp chromatin fragments. Sheared chromatin (approximately 5–10 µg) was incubated with 2 μg of anti-H3K4me1 (ab8895; Abcam) and H3K36me2 (ab9049; Abcam) ChIP-grade antibodies and normal rabbit IgG (#2729; CST) antibody at 4 °C (overnight). After overnight incubation, complexes with 30 μl of ChIP-Grade Protein G Magnetic Beads were incubated for 2 h at 4 °C. Then, the complexes were washed for each step, incubated with ChIP elution buffer for 30 min at 65 °C and then incubated with proteinase K for 2 h at 65 °C. After DNA purification using spin columns, the samples were analyzed by quantitative PCR using SMAD3 Primer. The primers were as follows: SMAD3 promoter region (P1) forward, 5′-GAGTGTGGACTCCGAGAGC-3′ and reverse, 5′-GCAGTCCTGGCTGGAGC-3′; SMAD3 (P2) forward, 5′-GCTCCAGCCAGGACTGC-3′ and reverse, 5′-CTTTCCAAGTGCTGTCACCG-3′; SMAD3 gene body region (P1) forward, 5′-CTCTTTGCGCACAGCTCT-3′ and reverse, 5′-AATGACTGCACCAAGGCC-3′; (P2) forward, 5′-CATAGAGGAGCAGCGTGACC-3′ and reverse, 5′-TCACTCCCCGCCTCTGC-3′; (P3) forward, 5′-GAGTGAGCTGAGGGCCAG-3′ and reverse, 5′-GCCCATTTCTCCCTGCAGA-3′; (P4) forward, 5′-GCTTTGCCGTCAAAGACTGC-3′ and reverse, 5′-GCTCTAAGAGGAACGCAGCA-3′; and (P5) forward, 5′-TGCTGCGTTCCTCTTAGAGC-3′ and reverse, 5′-GCTACCCGCAAAGGATCT G-3′.

### RNA-seq and analysis

Using the TruSeq RNA Sample Preparation Kit V2, purification and library construction were carried out with total RNA, and Illumina HiSeq 2500 instruments (Illumina, San Diego, CA, USA) were used for sequencing with a read length of 2 × 100 bases. FastQC v.0.11.4 was used for the quality of the paired-end reads. Cutadapt v.1.15 and Sickle v. 1.33 was used for filtering low-quality reads and adaptors. Cufflinks version 2.2.1 was used for calculation of fragments per kilobase of transcripts per million mapped reads (FPKM) values. Cuffdiff was used to select differentially expressed genes (DEGs) (fold change > 2)^26^. All Gene Ontology (GO) and KEGG pathway enrichment analyses were performed with the Database for Annotation, Visualization and Integrated Discovery (DAVID) ver. 6.8 and ClueGO ver. 2.5.5 in Cytoscape ver. 3.7.1.

### Animal experiments

Seven-week-old female NOD/SCID mice were purchased (GHBIO Inc.) for use in spleen injection experiments. For the liver metastasis experiments, 2 × 10^6^ H1299-luc shCont or shSMYD2 cells were resuspended in 100 μL Hanks’ balanced salt solution (HBSS) and injected into mouse spleens using a 30 G needle. After 1 min, the spleen was removed by the surgical procedure. Likewise, 10-week-old female NOD/SCID mice were used for the tail vein injection assay. A total of 1 × 10^6^ of the indicated cells were injected into the mouse tail vein using standard procedures (H1299-luc shCont or shSMYD2). All animal experimental protocols were approved by the Ethics Committees on Animal Experimentation of the Korea Research Institute of Bioscience and Biotechnology. For metastatic tumor imaging, in vivo bioluminescent imaging was performed with an IVIS Lumina III instrument (IVIS® Lumina III In Vivo Imaging System, PerkinElmer). Mice were first inoculated, and then 150 mg/kg D-luciferin was intraperitoneally injected as a luminescence substrate, and photon emission was detected in anesthetized (2% isoflurane) animals 5 min before image acquisition. Regions of interest from captured images were analyzed based on the tumor sites and quantified as total photon counts with Living Image® software (PerkinElmer).

### Public datasets of NSCLC patients

To validate the prognostic value of SMYD2, we used two independent cohorts of patients with NSCLC, all of whom had publicly available gene expression data: GSE8894^27^ is available in the NCBI GEO (https://www.ncbi.nlm.nih.gov/geo/), and the MSKCC dataset^28^ is available at http://cbio.mskcc.org/public/lung_array_data/.

### Statistical analysis

To classify patients into two groups, we performed receiver operating characteristic (ROC) analysis based on the gene expression value of SMYD2 and calculated the best cutoff value, defined as the point with the highest combined sensitivity and specificity. The Kaplan–Meier method was used to calculate the time to recurrence or to metastasis, and differences between the times were assessed using log-rank tests.

## Results

### SMYD2 is overexpressed in lung cancer

**Fig. 1:**
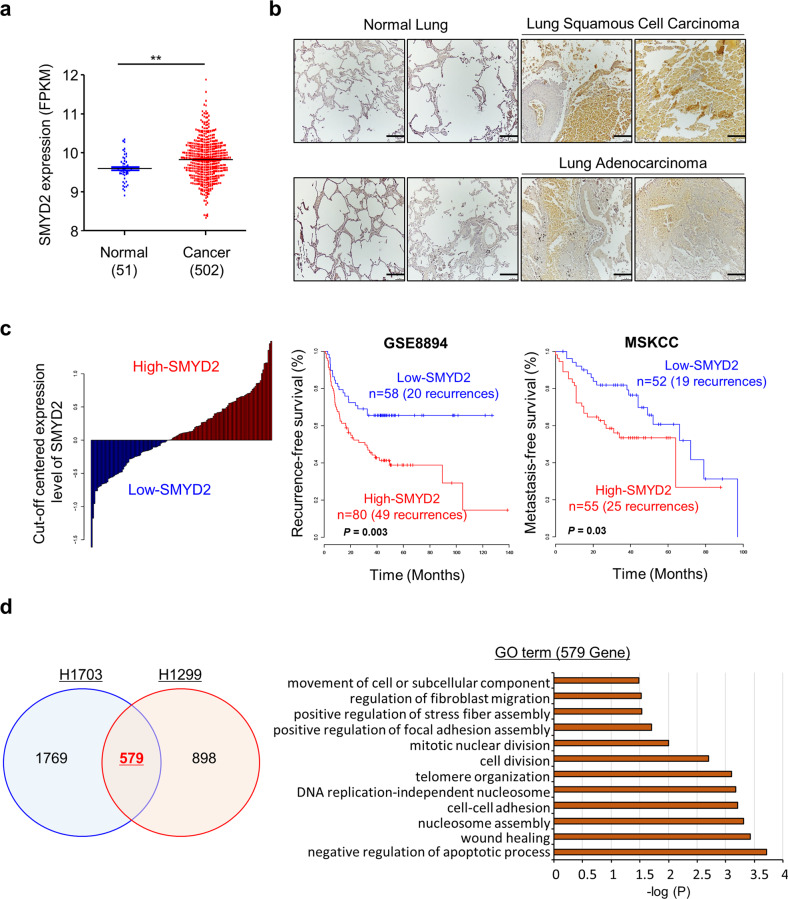
SMYD2 is overexpressed in lung cancer. **a** SMYD2 expression in normal and LUSC samples derived from the TCGA portal. *p* values were calculated using Student’s *t* *test* (**p < 0.01).  **b** Immunohistochemical analysis of SMYD2. Lung tumor and normal tissue arrays were purchased from BioChain (https://www.biochain.com). Scale bar, 200 μm. **c** Expression of SMYD2 and prognosis of NSCLC. The patients with NSCLC were divided into high- or low-expression SMYD2 subgroups according to the level of SMYD2 expression. In both patient cohorts (GSE8894 and MSKCC), Kaplan–Meier curves showed that the rates of recurrence or metastasis among patients with high SMYD2 expression were significantly higher than those among patients with low SMYD2 expression. **d** Identification of 579 DEGs that overlapped between the two cell lines (H1703 and H1299) and DAVID-based gene ontology analysis of RNA-seq results from the siSMYD2 (#1) and siCont groups.

To assess SMYD2 expression in lung cancer, we analyzed the RNA-seq results in normal lung (*n* = 51) and lung cancer (*n* = 502) samples derived from The Cancer Genome Atlas (TCGA) portal and found that SMYD2 was overexpressed at the transcriptional level (Fig. 1a). Additionally, immunohistochemical analysis of lung cancer and normal lung tissues showed that SMYD2 expression was increased in lung cancer tissues (adenocarcinoma and squamous cell carcinoma) compared to normal tissues (Fig. 1b). Moreover, in the patient cohort analysis, patients with high SMYD2 expression had a poor prognosis in terms of recurrence- and metastasis-free survival, implying that SMYD2 function is strongly related to lung cancer proliferation and metastasis (Fig. 1c).

Next, to assess the function of SMYD2, we performed RNA-seq analysis after transfection of lung cancer cell lines (H1703 and H1299) with siSMYD2 and siCont. In the comparison of siSMYD2 to siCont, 579 DEGs that overlapped between two cell lines (H1299 and H1703) were selected (Fig. 1d; left). The GO analysis of the 579 DEGs in DAVID version 6.8 (https://david.ncifcrf.gov/) showed multiple enriched cell migration-related GO terms (i.e., wound healing, regulation of fibroblast migration, cell–cell adhesion, and focal adhesion assembly) (Fig. 1d; right). Thus, on the basis of the GO term and cohort analysis results, we suggest that SMYD2 may be involved in lung cancer progression, especially metastasis.

### SMYD2 knockdown suppresses the migration and invasion of lung cancer cell lines

**Fig. 2:**
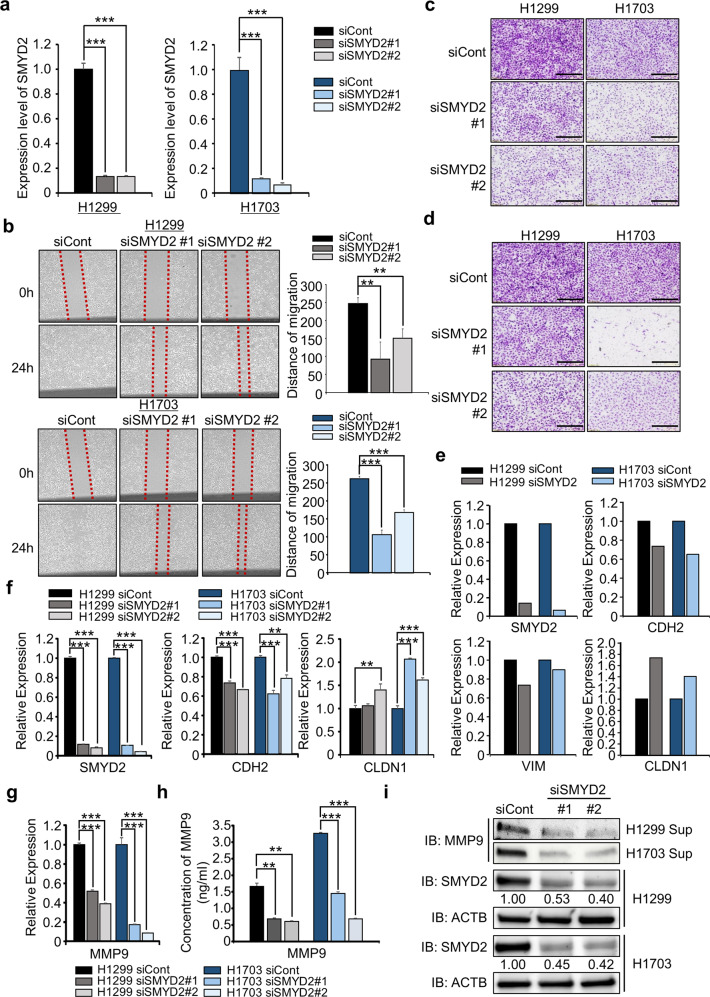
SMYD2 knockdown regulates the migration and invasion of lung cancer cell lines. **a** qRT–PCR analysis of SMYD2 after treatment of the H1299 (left) and H1703 (right) cell lines with SMYD2 (#1, #2) siRNA and siCont (negative control). The mean ± SD of three independent experiments is presented. *p* values were calculated using Student’s *t test* (***p < 0.001). **b** Wound healing assay. After 24 h of SMYD2 knockdown, scratch assays of the H1299 (upper) and H1703 (lower) cell lines were performed. After 24 h, wound closure was measured. The mean ± SD of three independent experiments is presented. *p* values were calculated using Student’s *t test* (**p < 0.01, ***p < 0.001). **c**, **d** Migration **c** and invasion **d** assays after SMYD2 knockdown in H1299 (left) and H1703 cell lines (right). Cell migration and invasion assays were performed after 36 h. Migrated/invaded cells were stained with crystal violet. Scale bar, 200 μm. **e** RNA-seq results from the siSMYD2 and siCont groups. Upregulation of epithelial cell markers (CLDN1) and downregulation of mesenchymal cell markers (CDH2, VIM). **f** qRT–PCR analysis of EMT markers (CDH2, CLDN1) after treatment with SMYD2 (#1, #2) siRNA and siCont in H1299 and H1703 cell lines. ACTB was used as an internal control. The mean ± SD of three independent experiments is presented. *p* values were calculated using Student’s *t test* (***p < 0.001, **p < 0.01). **g** qRT–PCR analysis of MMP-9 after treatment with SMYD2 (#1, #2) siRNA and siCont in H1299 and H1703 cell lines. The mean ± SD of three independent experiments is presented. *p* values were calculated using Student’s *t test* (***p < 0.001). **h** Decrease in MMP-9 concentration in the cell culture media using an MMP-9 ELISA kit after treatment of the H1299 and H1703 cell lines with SMYD2 (#1, #2) siRNA and siCont. The MMP-9 ELISA kit was purchased from Abcam. The mean ± SD of three independent experiments is presented. *p* values were calculated using Student’s *t test* (**p < 0.01, ***p < 0.001). **i** western blot analysis of MMP-9 in cell culture media using anti-MMP-9 antibody after treatment of the H1299 and H1703 cell lines with SMYD2 (#1, #2) siRNA and siCont. ACTB was used as the internal control in H1299 and H1703 cells. The signal intensities were quantified using ImageJ software.

Based on the GO terms, we first designed SMYD2-specific siRNA (siSMYD2) and control siRNA (siCont) (see Materials and Methods) and transfected them into two lung cancer cell lines (H1299 and H1703). qRT–PCR analysis showed that the expression of SMYD2 was significantly suppressed by siSMYD2 compared to siCont (Fig. 2a). Next, after siRNA transfection, we performed a wound healing assay. Figure 2b shows that the rates of wound closure were higher in the siCont groups than in the siSMYD2 groups of H1299 and H1703 cells. Additionally, in the migration and invasion analysis, SMYD2 knockdown inhibited cell migration and invasion compared to that in the siCont group (Fig. 2c, d). To verify SMYD2-related cell migration/invasion, we assessed the expression of EMT markers (the epithelial marker CLDN1 and mesenchymal markers CDH2 and VIM) by RNA-seq after transfection with siSMYD2. We observed downregulation of mesenchymal markers and upregulation of epithelial markers (Fig. 2e). In the qRT–PCR analysis, we observed the same trends in EMT marker expression between the siSMYD2 and siCont groups (Fig. 2f).

Matrix metalloproteinase-9 (MMP-9) is a protease that can cleave extracellular matrix (ECM) proteins to remodel the ECM and is related to angiogenesis, metastasis, and invasion^29–31^. Because SMYD2 knockdown suppressed cell invasion, we evaluated the expression level of MMP-9 after SMYD2 knockdown and found significant suppression of MMP-9 expression at the transcriptional level (Fig. 2g). Moreover, we verified the decrease in MMP-9 concentration by using an MMP-9 ELISA detection kit in the cell culture media (Fig. 2h) and detected a reduction in MMP-9 expression in the supernatants of the siSMYD2 groups compared to that of the siCont groups by western blot analysis (Fig. 2i). Together, these data suggest that SMYD2 may be a regulator of lung cancer metastasis.

### SMAD3 is a direct target of SMYD2 in lung cancer

**Fig. 3:**
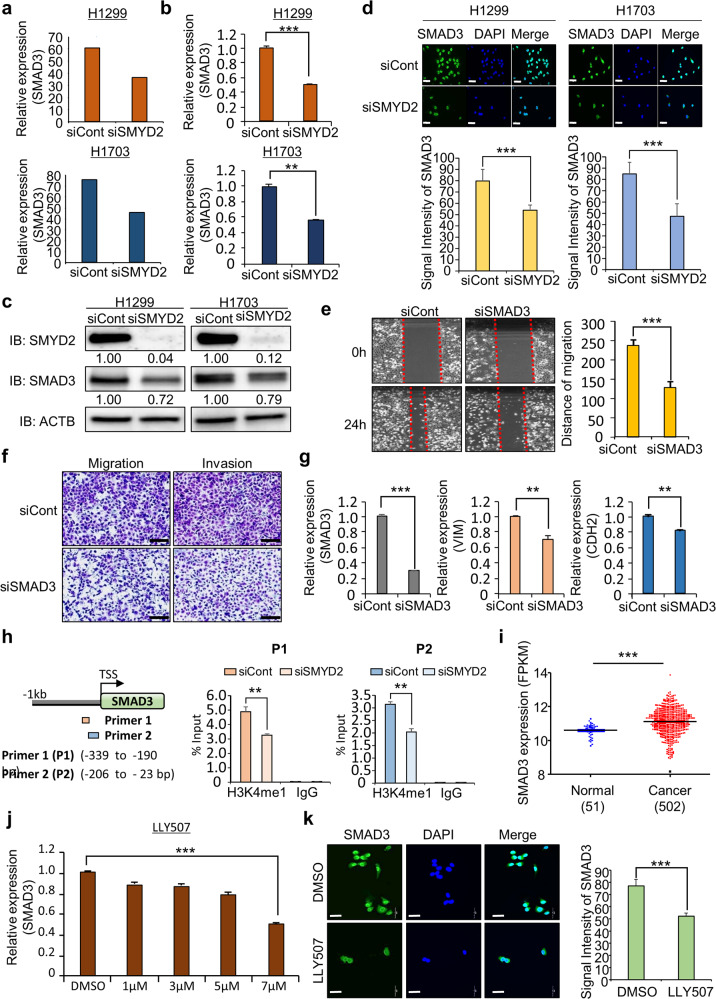
SMAD3 is directly regulated by SMYD2-related epigenetic regulation. **a** RNA-seq analysis of SMAD3 after treatment with SMYD2 siRNA (#1) and siCont in H1299 (upper) and H1703 (lower) cell lines. **b** qRT–PCR analysis of SMAD3 after treatment with SMYD2 siRNA (#1) and siCont in H1299 (upper) and H1703 (lower) cell lines. The mean ± SD of three independent experiments is presented. *p* values were calculated using Student’s *t test* (***p < 0.001, **p < 0.01). **c** Western blot analysis of SMAD3 after treatment of the H1299 (left) and H1703 (right) cell lines with SMYD2 siRNA (#1) and siCont. ACTB was used as the internal control. The signal intensities were quantified using ImageJ software. **d** Immunocytochemical analysis of SMAD3. H1299 (left) and H1703 (right) cells treated with siSMYD2 (#1) were fixed with 100% methanol and stained with anti-SMAD3 (Alexa Fluor 488, green) and DAPI (blue) (upper). Quantification of SMAD3 expression in the immunocytochemical analysis. The mean ± SD of three independent experiments is presented. *p* values were calculated using Student’s *t test*s (***p < 0.001) (below). Scale bar, 200 μm. **e** Wound healing assay. After 24 h of SMAD3 knockdown, scratch assays of H1299 cells were performed. After 24 h, wound closure was measured. The mean ± SD of three independent experiments is presented. *p* values were calculated using Student’s *t test* (***p < 0.001). **f** Migration (left) and invasion (right) assays after SMAD3 knockdown in H1299 cells. Cell migration and invasion assays were performed after 36 h. Migrated/invaded cells were stained with crystal violet. Scale bar, 200 μm. **g** qRT–PCR analysis of EMT markers (VIM, CDH2) after treatment of H1299 cells with SMAD3 siRNA and siCont. The mean ± SD of three independent experiments is presented. *p* values were calculated using Student’s *t test* (***p < 0.001, **p < 0.01). **h** Graphical abstract for ChIP primer design on the SMAD3 promoter region. ChIP assays were performed using an anti-H3K4 monomethylation antibody and normal IgG antibody on the SMAD3 promoter region. **i** SMAD3 expression in normal and LUSC samples derived from the TCGA portal. *p* values were calculated using Student’s *t* *test* (***p < 0.001). **j** qRT–PCR analysis of SMAD3 expression levels according to LLY507 concentration (DMSO or 1, 3, 5, or 7 µM). The mean ± SD of three independent experiments is presented. *p* values were calculated using Student’s *t test* (***p < 0.001). **k** Immunocytochemical analysis of SMAD3. H1299 cells treated with DMSO or 7 µM LLY507 were fixed with 100% methanol and stained with anti-SMAD3 (Alexa Fluor 488, green) and DAPI (blue) (left). Quantification of SMAD3 expression in the immunocytochemical analysis. The mean ± SD of three independent experiments is presented. *p* values were calculated using Student’s *t test*s (***p < 0.001) (right). Scale bar, 200 μm.

Histone methylation by SMYD2 is involved in the activation of gene transcription via the epigenetic machinery^32^. Thus, we performed a candidate approach to identify the direct target of SMYD2 among the downregulated genes in the RNA-seq analysis and finally selected SMAD3 (Fig. 3a). Notably, the expression of SMAD2 and SMAD4 was not reduced by SMYD2 knockdown according to the RNA-seq results and qRT–PCR analysis (Supplementary Fig. 1). Thus, we focused on the SMYD2-SMAD3 axis and hypothesized that it may be involved in lung cancer metastasis. We validated the downregulation of SMAD3 by SMYD2 knockdown in lung cancer cell lines by qRT–PCR analysis (Fig. 3b). Additionally, we confirmed SMAD3 downregulation by SMYD2 knockdown via western blotting and immunocytochemical analysis (Fig. 3c, d). Next, to determine whether SMAD3 downregulation affects cell migration and invasion, we performed a wound healing assay after SMAD3 knockdown and observed a reduction in the wound closure speed compared to that in the siCont group (Fig. 3e). Moreover, SMAD3 knockdown suppresses the migration and invasion of lung cancer cell line, and the expression of VIM and CDH2 was decreased by SMAD3 knockdown (Fig. 3f, g). Thus, we suggest that downregulation of SMAD3 by SMYD2 knockdown strongly affects the migration and invasion of lung cancer cell lines.

Next, we performed a ChIP assay to validate SMAD3 as a direct target of SMYD2 using primers targeting the SMAD3 promoter region (Fig. 3h; left). SMYD2 knockdown reduced the status of H3K4 monomethylation compared to siCont (Fig. 3h; right). Because SMYD2 mainly methylates the H3K36 histone demethylase in the SMAD3 gene body^33^, we performed a ChIP assay using an anti-H3K36 dimethylation antibody. However, we did not observe a change in the H3K36 dimethylation status in the terminal region of the SMAD3 gene body (Supplementary Fig. 2). Thus, SMYD2 knockdown reduced the methylation of its direct target, SMAD3, via a consequent reduction in H3K4 monomethylation. Moreover, in the TCGA data, we found upregulation of SMAD3 in lung cancer samples compared to normal samples, as shown by SMYD2 upregulation (Fig. 3i), suggesting that SMAD3 regulation by SMYD2 could regulate lung cancer metastasis.

### The SMYD2-specific inhibitor LLY507 reduces SMAD3 expression

LLY507 is a specific inhibitor that reduces SMYD2 activity^34,35^. After treatment of the H1299 cell line with LLY507, we observed clear reductions in wound healing and cell migration/invasion ability (Supplementary Fig. 3a-b). Moreover, the expression of EMT markers was changed by LLY507 treatment compared to DMSO treatment, similar to siSMYD2 transfection (Supplementary Fig. 3c). Additionally, SMAD3 expression at the transcriptional level decreased in a dose-dependent manner in the LLY507 treatment group compared to the DMSO group (Fig. 3j). Consistently, western blot analysis showed that SMAD3 expression was decreased by LLY507 treatment (Supplementary Fig. 3d). Similarly, we observed a reduction in SMAD3 expression in the LLY507 treatment group via immunocytochemical analysis (Fig. 3k). Thus, inhibition of SMYD2 with a specific inhibitor suppressed lung cancer metastasis, and these results suggest that the development of an SMYD2-specific inhibitor might be important for lung cancer treatment.

### Suppression of the lung metastasis signature by SMYD2 knockdown in invasive H1299 cell lines

**Fig. 4:**
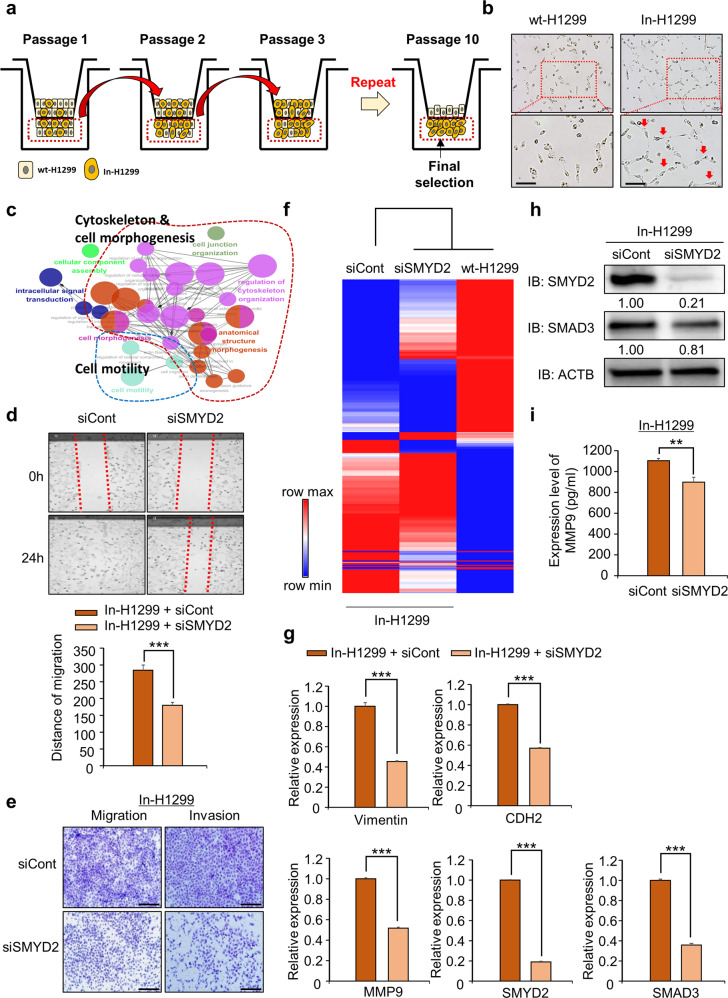
SMYD2 knockdown suppresses the migration and invasion of highly invasive H1299 cell lines. **a** Experimental scheme for establishing In-H1299 cells. **b** Microscopic image of wt-H1299 or In-H1299 phenotypes. Scale bar, 200 μm. **c** GO pathway term enrichment networks. GO pathway term networks in the wt-H1299 and In-H1299 groups were functionally grouped by ClueGO. **d** Wound healing assay. After 24 h of SMYD2 knockdown, scratch assays of In-H1299 cells were performed. After 24 h, wound closure was assessed. The mean ± SD of three independent experiments is presented. *p* values were calculated using Student’s *t test* (***p < 0.001). **e** Migration (left) and invasion (right) assays after SMYD2 knockdown in In-H1299 cells. Cell migration and invasion assays were performed after 24 h. Migrated/invaded cells were stained with crystal violet. Scale bar, 200 μm. **f** Heatmap of the RNA-seq results for siCont and siSMYD2 in In-H1299 cells. **g** qRT–PCR analysis of EMT markers (VIM, CDH2, MMP-9) and SMAD3 expression levels after transfection with SMYD2 siRNA and siCont in In-H1299 cells. The mean ± SD of three independent experiments is presented. *p* values were calculated using Student’s *t test* (***p < 0.001). **h** Western blot analysis of SMAD3 after treatment with SMYD2 siRNA and siCont in In-H1299 cells. ACTB was used as the internal control. The signal intensities were quantified using ImageJ software. **i** Decrease in MMP-9 concentration in the cell culture media using an MMP-9 ELISA kit after treatment of In-H1299 cells with SMYD2 siRNA and siCont. The MMP-9 ELISA kit was purchased from Abcam. The mean ± SD of three independent experiments is presented. *p* values were calculated using Student’s *t test* (**p < 0.01).

To assess whether SMYD2 knockdown could affect the metastatic signature of invasive lung cancer cells, we generated highly invasive H1299 cell lines via 10 repeated migration experiments, as shown in Fig. 4a. Using a Transwell system, Tie et al. previously reported that gastric cancer cell lines acquired highly invasive characteristics after EMT in vitro^36^. Using our modified in vitro EMT system, we observed highly invasive H1299 cell lines. Examination by bright field microscopy revealed that the invasive H1299 cell line (In-H1299) appeared to have a more mesenchymal phenotype than wild-type H1299 (wt-H1299) cells (Fig. 4b). Moreover, we detected clear upregulation of CDH2 and MMP-9 and downregulation of CLDN1, implying that In-H1299 cells become more invasive than wt-H1299 cells (Supplementary Fig. 4a). Wound healing analysis showed that the rate of wound closure for In-H1299 cells was higher than that for wt-H1299 cells (Supplementary Fig. 4b). Migration and invasion assays showed that the capacity for migration and invasion was significantly increased in In-H1299 cells compared to wt-H1299 cells (Supplementary Fig. 4c). Moreover, the concentration of MMP-9 in the culture media of In-H1299 cells was higher than that in the media of wt-H1299 cells (Supplementary Fig. 4d). We combined the in vitro EMT system with a cell migration system to generate highly invasive H1299 cell lines for further study. In the GO term analysis (ClueGO) of the RNA-seq results for wt-H1299 and In-H1299 cell lines, migration-related terms were enriched in In-H1299 cells compared to wt-H1299 cells (Fig. 4c). Interestingly, the expression of SMYD2 and SMAD3 was increased in In-H1299 cells, suggesting that SMYD2 may be related to the metastatic signature of invasive lung cancer (Supplementary Fig. 4e).

Next, using highly invasive H1299 cell lines (In-H1299 cells), we assessed the effect of SMYD2 knockdown. After treating In-H1299 cells with siSMYD2, we performed wound healing and migration/invasion assays. As shown in Fig. 4d, e, we observed that the wound closure speed and migration/invasion activity were clearly significantly suppressed by SMYD2 knockdown compared to siCont transfection, suggesting that SMYD2 knockdown effectively inhibited metastatic activity in In-H1299 cells as well as in wt-H1299 cells. In the heatmap analysis with RNA-seq results for siCont and siSMYD2 in In-H1299 cells, clustering of samples was performed by first clustering siSMYD2 and wt-H1299 compared to siCont samples (Fig. 4f). Regarding the molecular pathway, we observed SMAD3 downregulation by SMYD2 knockdown in In-H1299 cells, as observed in wt-H1299 cells, and the expression of CDH2, VIM and MMP-9 was significantly decreased in the qRT–PCR analysis (Fig. 4g). Moreover, western blot analysis showed that the expression of SMAD3 was reduced by siSMYD2 transfection in In-H1299 cells (Fig. 4h). Additionally, the concentration of MMP-9 in the culture media of H1299 cells was decreased by siSMYD2 transfection (Fig. 4i). Thus, we suggest that SMYD2 downregulation inhibits the metastasis of invasive lung cancer and primary lung cancer.

### SMYD2 knockdown inhibits lung cancer metastasis in vivo

**Fig. 5:**
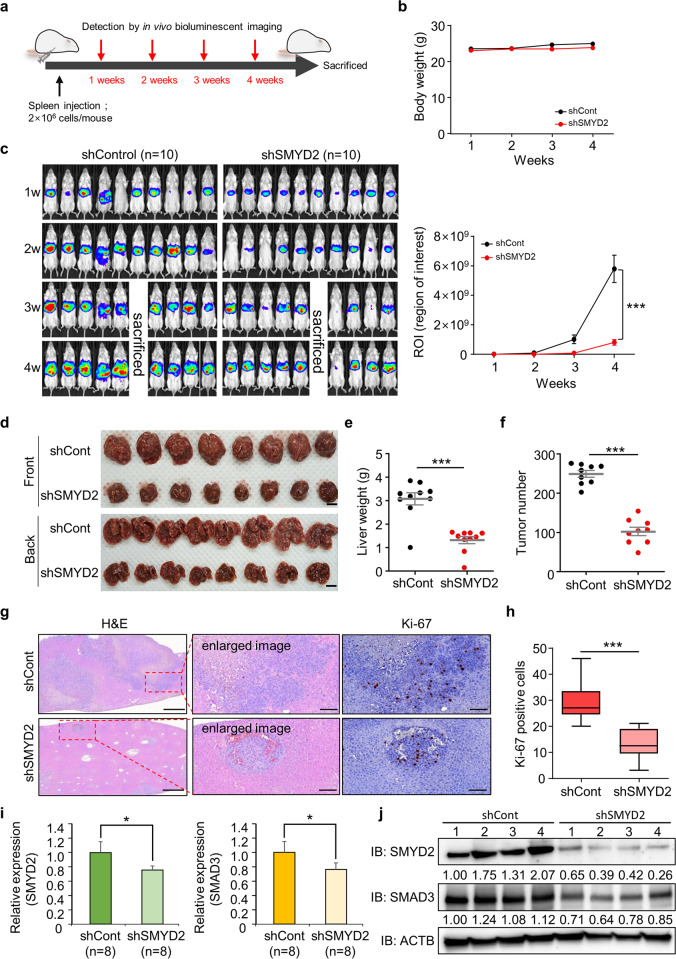
SMYD2 is a key regulator of liver metastasis. **a** Scheme of spleen transplantation of H1299-Luc shCont or shSMYD2 cells. **b** Body weight of mice after the spleen injection of H1299-Luc shCont or shSMYD2 cells. **c** IVIS images were obtained once a week until 4 weeks after splenic injection of H1299-Luc shCont or shSMYD2 cells (left) and the average luminescence intensity of photons emitted from tumors (right). *p* values were calculated using Student’s *t* *test* (***p < 0.001). **d** Macroscopic images of whole livers from mice at 4 weeks after spleen transplantation with shCont H1299 cells and shSMYD2-expressing H1299 cells. **e**, **f** Liver weight **e** and tumor number in H1299-Luc shCont- and shSMYD2-injected mouse livers **f**. **g** Representative H&E staining (left), enlarged images of H&E (middle) and Ki-67 immunostaining (right) of H1299-Luc shCont- and shSMYD2-injected mouse liver sections. Scale bars, 200 μm (left) and 50 μm (middle, right). **h** Ki-67-positive cells in shCont and shSMYD2 liver sections. *p* values were calculated using Student’s *t* *test* (***p < 0.001). **i** qRT–PCR analysis of SMYD2 and SMAD3 expression levels in liver metastatic tumors. The mean ± SD of three independent experiments is presented. *p* values were calculated using Student’s *t test* (*p < 0.05). **j** Western blot analysis of SMAD3 and SMYD2 in liver metastatic tumors. ACTB was used as the internal control. The signal intensities were quantified using ImageJ software.

To verify the function of SMYD2 in lung cancer, we constructed shSMYD2 and shCont H1299 cell lines under puromycin selection. First, we performed a wound healing assay using the shSMYD2 and shCont H1299 cell lines and observed clear wound closure in shCont cells compared to shSMYD2 H1299 cells (Supplementary Fig. 5a). Additionally, in the migration and invasion assays, the number of shSMYD2 H1299 cells was decreased compared to that of shCont H1299 cells (Supplementary Fig. 5b). Regarding EMT marker expression, CDH2 and VIM were decreased, and CLDN1 was increased in shSMYD2 H1299 cells, as observed in siSMYD2-transfected cells (Supplementary Fig. 5c). Moreover, we observed the downregulation of SMAD3 expression in shSMYD2 H1299 cells by qRT–PCR analysis and western blot analysis (Supplementary Fig. 5d, e). Additionally, we confirmed SMAD3 downregulation in shSMYD2 H1299 cells via immunocytochemical analysis (Supplementary Fig. 5f). Next, using the shSMYD2 and shCont H1299 cell lines, we performed an in vivo study via spleen injection to observe liver metastasis. The shCont or shSMYD2 H1299-luc cells, including the luciferase gene, were injected into the mouse spleen, and then the body weight and luciferase activity were measured by using IVIS imaging (Fig. 5a). There was no significant difference in body weight between the shCont and shSMYD2 groups (Fig. 5b), and we found a marked reduction in luciferase activity in the shSMYD2 group compared with the shCont group (Fig. 5c). Moreover, consistent with the luciferase activity results, the liver weight and nodule number of resected liver tissues were also significantly decreased in the shSMYD2 group (Fig. 5d–f). In H&E staining and Ki-67 immunohistochemical analysis of liver tissue, we also observed that the proliferating cells in the tumor region were significantly decreased in the shSMYD2 group (Fig. 5g, h). Finally, to assess the expression levels of SMYD2 and SMAD3, we performed qRT–PCR and western blot analysis of liver metastasis tissues. Figure 5i, j shows that the expression levels of SMYD2 and SMAD3 decreased at the transcriptional and translational levels, implying that downregulation of SMAD3 by SMYD2 affects lung cancer metastasis in vivo.

**Fig. 6:**
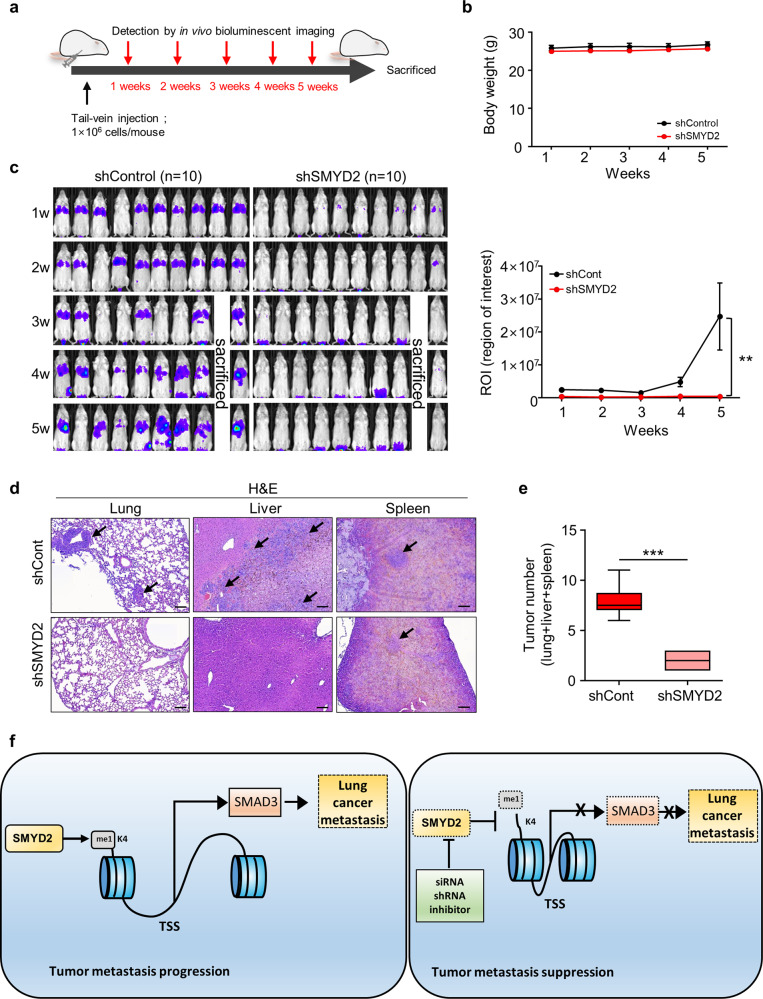
SMYD2 silencing decreased in vivo metastasis. **a** Scheme of the intravenous injection of H1299-Luc shCont or shSMYD2 cells. **b** Body weight of mice after intravenous injection of H1299-Luc shCont or shSMYD2 cells. **c** IVIS images were obtained once a week until 5 weeks after intravenous injection of H1299-Luc shCont or shSMYD2 cells and the average luminescence intensity of photons emitted from tumors. *p* values were calculated using Student’s *t* *test* (**p < 0.01). **d** Representative H&E staining of lung (left), liver (middle) and spleen (right) sections. Scale bar, 100 μm. Arrows indicate micrometastatic tumors. **e** Measurement of micrometastatic tumor numbers in the lung, liver and spleen by H&E staining. *p* values were calculated using Student’s *t test* (***p < 0.001). **f** Schematic summary of SMYD2-related lung cancer metastasis.

Next, to verify the in vivo results of SMYD2-dependent lung metastasis in more detail, we performed another metastasis experiment. The shCont or shSMYD2 H1299-luc cells were injected into the tail vein, and the tumors were followed up with an IVIS imaging system for 5 weeks (Fig. 6a). The body weight was not significantly different between the shCont and shSMYD2 groups (Fig. 6b), and the luciferase activity was significantly decreased in the shSMYD2 group compared with the shCont group in the lung (Fig. 6c). Histologically, we found that the metastatic tumor numbers in the lung, liver and spleen were significantly decreased in the shSMYD2 group (Fig. 6d, e). Taken together, these findings present SMYD2 as a regulator of lung cancer metastasis.

## Discussion

Cancer metastasis is estimated to be the main cause of cancer-related death, accounting for approximately 90% of such deaths. The EMT process promotes metastasis as it plays important roles in promoting cell migration and invasion and the acquisition of stem cell characteristics^37^. Thus, in the quest to decrease the mortality rate of cancer, new metastasis regulators have been recognized as a therapeutic option to reduce the macro- or micrometastasis of cancers. Here, we hypothesized that the SMYD2-SMAD3 axis influences the epigenetic regulation of the EMT process and found that in in vivo and in vitro models, SMYD2 knockdown clearly suppressed cell migration and invasion.

SMYD2 is responsible mainly for the methylation of H3K36 in the target gene body, which leads to euchromatin formation^32^, but SMYD2 can also perform H3K4 monomethylation in the promoter region of target genes to upregulate gene expression^9,38^. To verify the relationship between SMYD2 and SMAD3, we first checked the H3K36 dimethylation status in the terminal region of the SMAD3 gene in a ChIP assay, but we could not find significant results regarding the H3K36 dimethylation status in the terminal region of the SMAD3 gene body. Although we confirmed that SMYD2 directly regulates SMAD3 expression by observing whether there was a change in H3K4 monomethylation in the promoter region of SMAD3, it is difficult to say with certainty that the association between H3K36 methylation and SMAD3 regulation is irrelevant because we did not examine methylation of the entire gene body. Therefore, if ChIP-seq analysis is performed using an H3K36 dimethylation antibody in further studies, it is expected that it will be possible to explain the regulatory mechanism of SMAD3 on H3K36 dimethylation by SMYD2.

Among the majority of patients with various cancers, the survival rate is low due to metastasis^39,40^. To increase the therapeutic effect of cancer treatment, targeting and inhibiting specific genes and pathways, including (1) those involved in the EMT process in cancer cells and (2) those that are characteristic of aggressive cancer cells, which already have mesenchymal characteristics, would be effective for combating cancer metastasis. Thus, in this study, to verify the function of SMYD2 in highly invasive lung cancer, we generated invasive H1299 cell lines using a Transwell system. After 10 reiterations, we observed a clearly elongated cell shape, critical upregulation of MMP-9 expression and an increase in the migration/invasion rates (Fig. 4 and Supplementary Fig. 4). To construct an in vitro EMT system, TGF-β treatment and Transwell systems have been widely used^36,41^. SMADs 2, 3, and 4 are key transcriptional regulators of TGF-β signaling and are involved in the TGF-β-induced EMT process. During cancer metastasis, activation of the TGF-β pathway via secretion of TGF-β from the tumor microenvironment promotes the construction of the SMAD complex and upregulation of EMT-related genes for cancer metastasis^21^. To validate the effect of SMYD2 on the TGF-β-induced EMT process, we performed migration and invasion analyses after siSMYD2 treatment with TGF-β and observed that SMYD2 downregulation clearly reduced the number of migrated and invasive H1299 cells, as shown by in vitro EMT experiments using a Transwell system (Supplementary Fig. 6). Thus, we suggest that SMYD2 downregulation effectively suppresses cancer metastasis and that SMYD2 is a potential metastasis regulator for lung cancer.

In the in vivo study, although the spleen transplantation results were not significantly different between the shCont and shSMYD2 groups, the liver metastasis rate tended to decrease in mice injected with SMYD2 knockdown H1299 cells compared with those injected with shCont cells (Fig. 5e, f). Spleen transplantation experiments and tail vein injection studies usually show half of the metastasis cascade, including the extravasation and colonization steps. Our in vitro data showed that knockdown of SMYD2 decreased the expression of EMT-related genes (Fig. 2), indicating that loss of SMYD2 may decrease the efficiency of cancer cell penetration from vessels to the liver.

In conclusion, using the TCGA portal, we observed clear overexpression of SMYD2 in lung cancer tissues compared to normal tissues. Downregulation of SMYD2 suppressed the migration and invasion of lung cancer cell lines by reducing SMAD3 expression through SMYD2-mediated epigenetic regulation. Furthermore, in the highly invasive H1299 cell lines constructed with an in vitro EMT system, SMYD2 reduction suppressed metastasis-specific features, such as the expression of EMT markers and MMP-9. Additionally, the in vivo model confirmed that SMYD2 is related to lung cancer metastasis (Fig. 6f). Thus, SMYD2 is a potential anti-metastasis target for lung cancer treatment, suggesting that the incidence of micro/macrometastasis in lung cancer will be reduced by SMYD2-specific inhibitors.

## Supplementary information

Supplementary figures 1-6
