# Supplementary material for: Epigenetic regulation of SMAD3 by histone methyltransferase SMYD2 promotes lung cancer metastasis
Source: Exp Mol Med. 2023 May 1;55(5):952–64. doi: 10.1038/s12276-023-00987-1 (PMC10238379; doi:10.1038/s12276-023-00987-1)
Supplement: Supplementary file 1 — Supplementary figures 1-6 [file 12276_2023_987_MOESM1_ESM.pdf]

Epigenetic regulation of SMAD3 by histone methyltransferase SMYD2 promotes lung cancer metastasis

Kwangho Kim[1,6,7], Tae Young Ryu[1,7], Eunsun Jung[1,7], Tae-Su Han[1,2,7], Jinkwon Lee[1,2], Seon-Kyu Kim[1], Yu Na Roh[1,2], Moo-Seung Lee[1], Cho-Rok Jung[1,2], Jung Hwa Lim[1], Ryuji Hamamoto[3], Hye Won Lee[4], Keun Hur[5], Mi-Young Son[1,2], Dae-Soo Kim[1, 2], Hyun-Soo Cho[1, 2]

[1]Korea Research Institute of Bioscience and Biotechnology, Daejeon, 34141, Republic of Korea; [2]Department of Functional Genomics, Korea University of Science and Technology, Daejeon, 34316, Republic of Korea; [3]Division of Molecular Modification and Cancer Biology, National Cancer Center, Tokyo, 104–0045, Japan; [4] Department of pathology, Keimyung University School of Medicine, 42601, Republic of Korea; [5] Department of Biochemistry and Cell Biology, School of Medicine, Kyungpook National University, Daegu, 41944, Republic of Korea; [6]College of Pharmacy, Chungnam National University, Daejeon, 34134, Republic of Korea

[7]These authors contributed equally to this work

Supplementary figures: 1-6

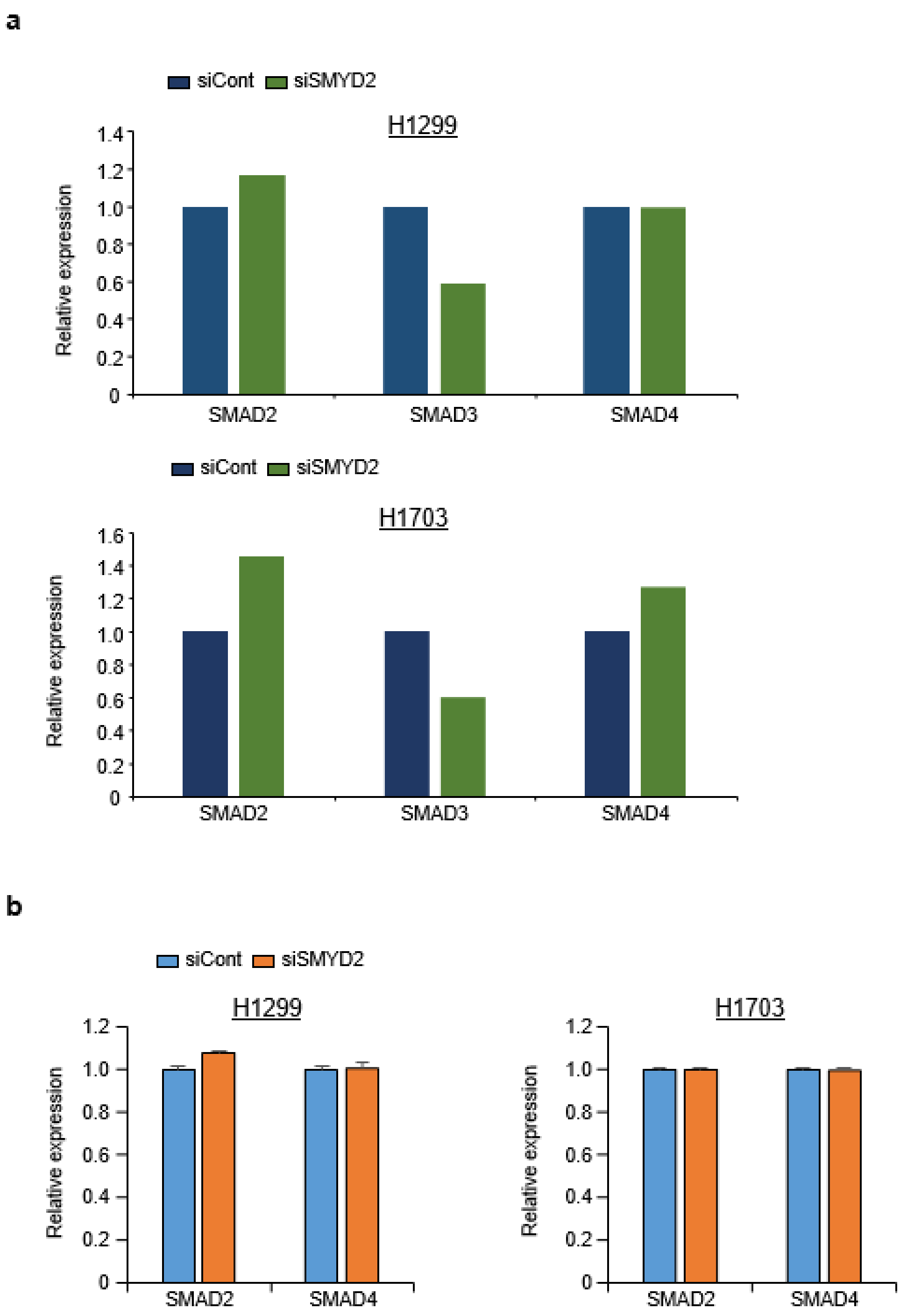

Supplementary Fig. 1. SMYD2 knockdown does not affect SMAD3 or SMAD4 expression. **a** RNA-seq analysis of SMAD2/3/4 after transfection of H1703 (left) and H1299 (right) cell lines with SMYD2 siRNA or siCont. **b** qRT-PCR analysis of SMAD2/4 expression levels after transfection of H1299 (left) and H1703 (right) cell lines with SMYD2 siRNA and siCont.

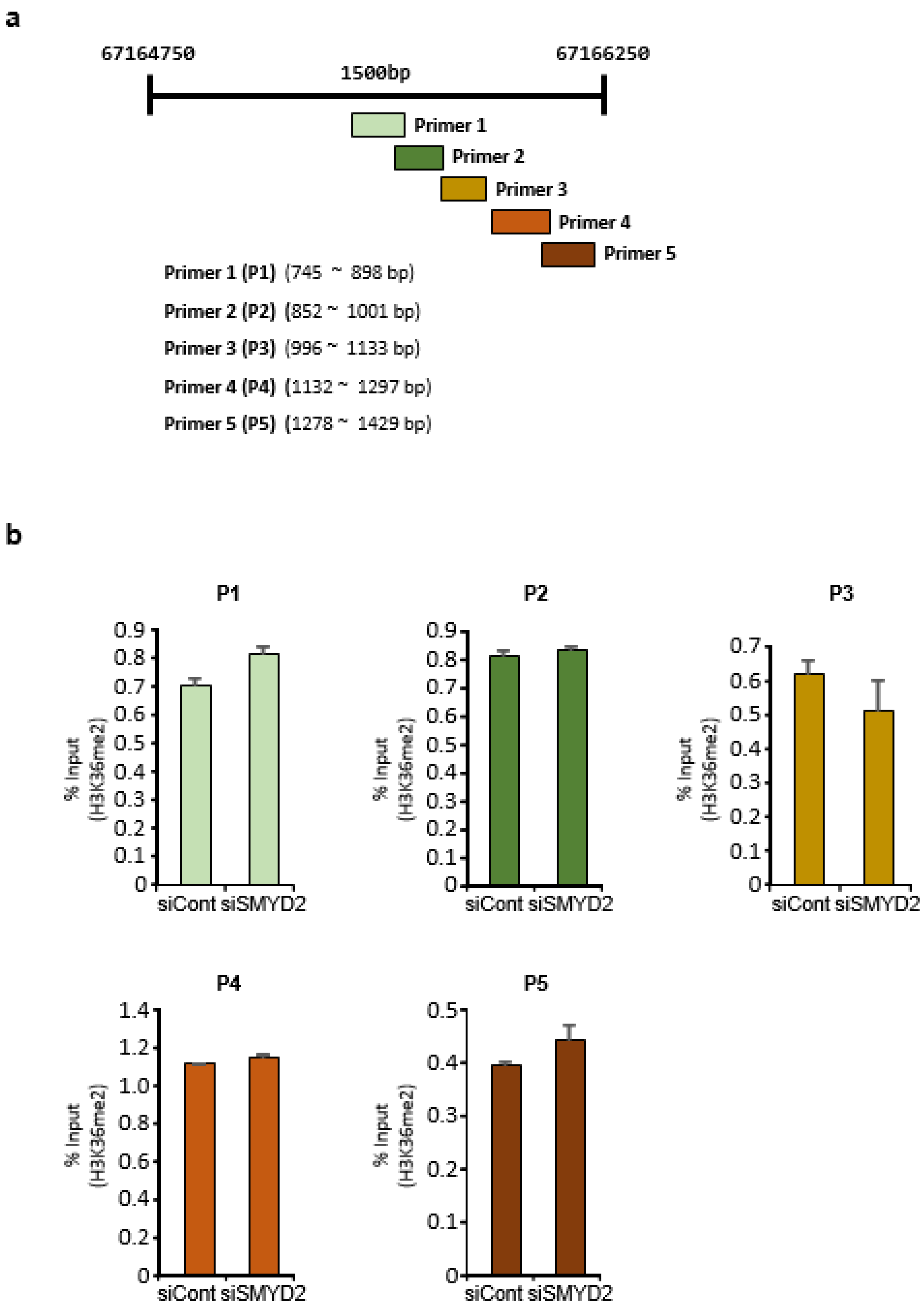

Supplementary Fig. 2. ChIP analysis with H3K36 dimethylation antibody after SMYD2 knockdown. a Graphical abstract for ChIP primer design on the SMAD3 gene body region. **b** The ChIP assay was performed using an anti-H3K36 dimethylation antibody. The result is shown as a percentage of input chromatin in H1299 cells.

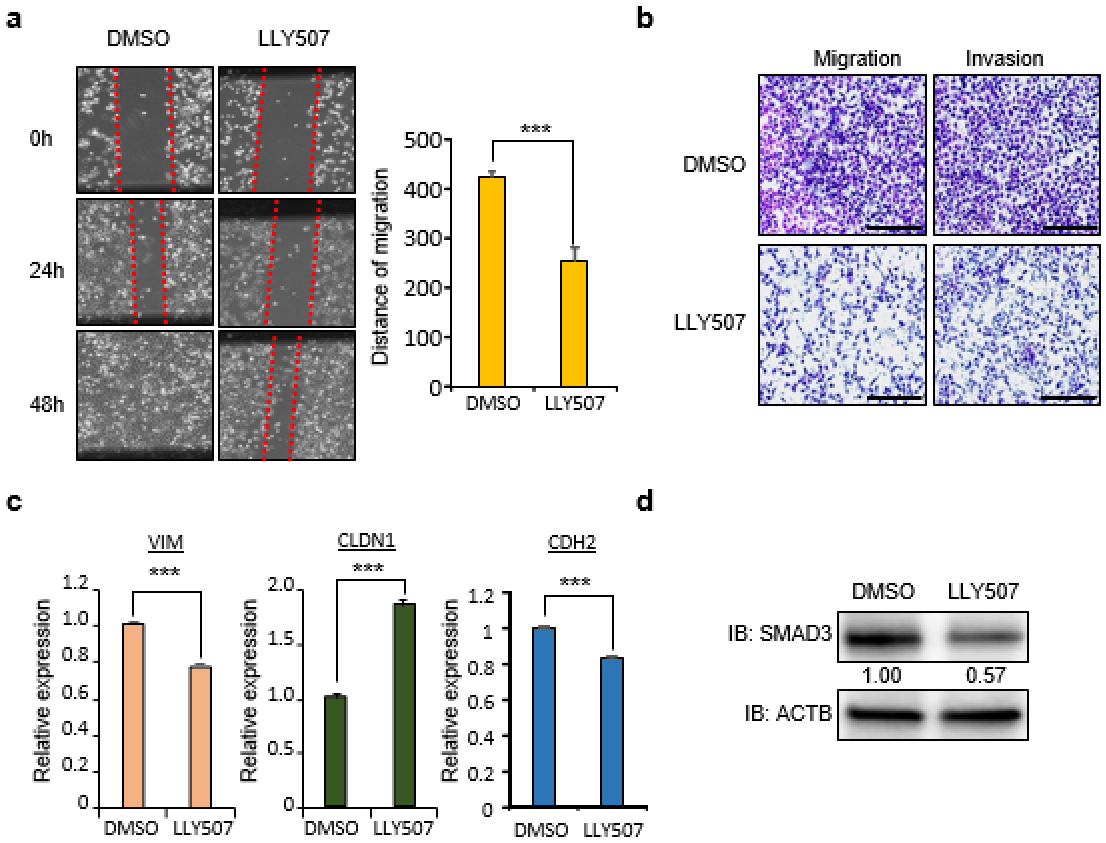

Supplementary Fig. 3. SMYD2-specific inhibitor (LLY507) suppresses lung cancer metastasis. a Wound-healing assay. After treatment with DMSO or LLY507 7 μM, scratch assays of H1299 cells were performed, and wound closure was measured after 24 h and 48 h. The mean ± SD of three independent experiments is presented. *p* values were calculated using Student's *t test* (****p* < 0.001). **b** Migration (left) and invasion (right) assays after treatment with DMSO or LLY507 7 μM in H1299 cells. Cell migration and invasion assays were performed after 36 h. Migrated/invaded cells were stained with crystal violet. Scale bar, 200 μm. **c** qRT–PCR analysis of EMT markers (VIM, CLDN1, CDH2) after treatment with DMSO or LLY507 7 μM in H1299 cells. The mean ± SD of three independent experiments is presented. *p* values were calculated using Student's *t test* (****p* < 0.001). **d** Western blot analysis of SMAD3 after treatment of H1299 cells with DMSO or LLY507 7 μM. ACTB was used as the internal control. The signal intensities were quantified using ImageJ software.

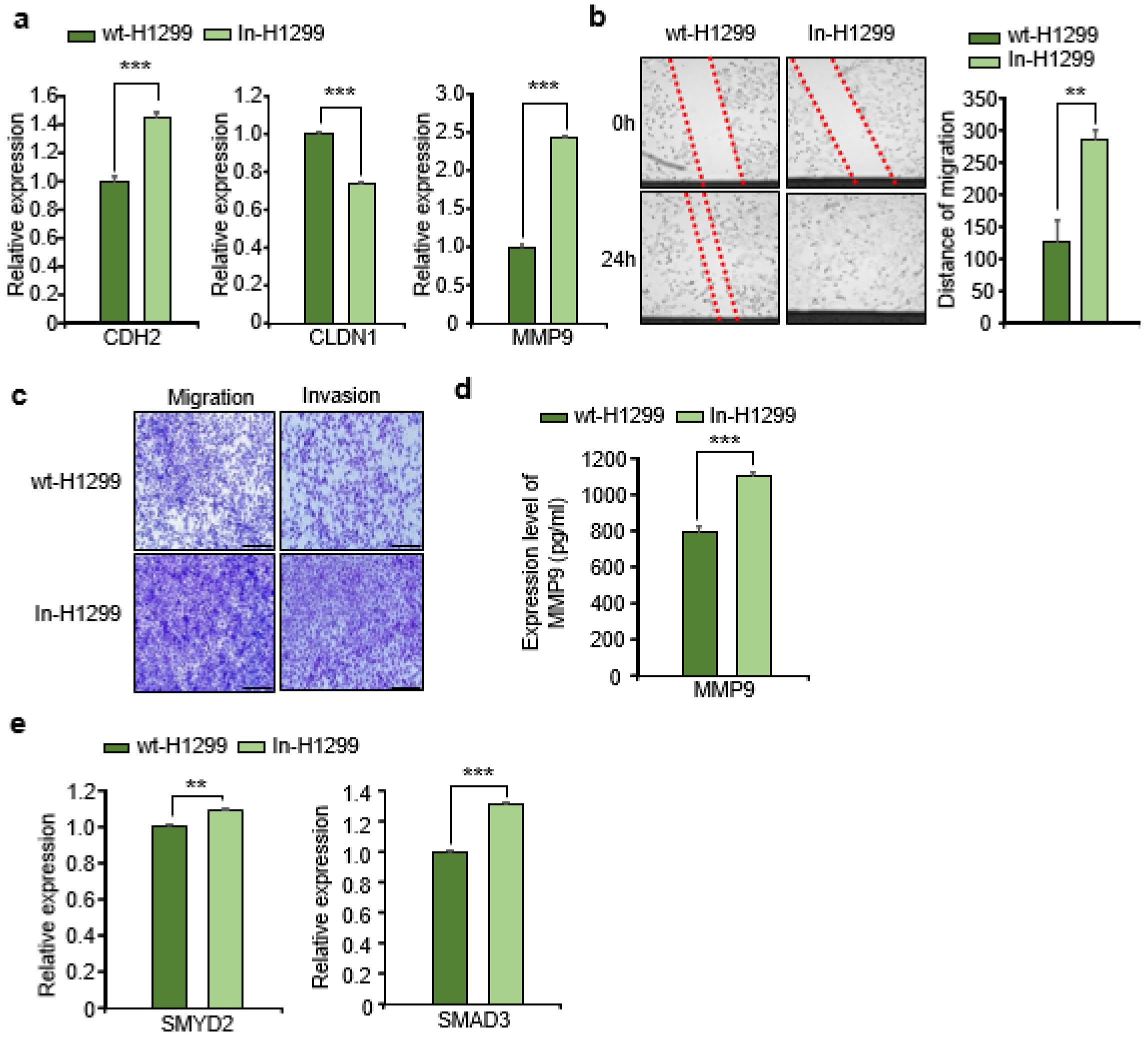

Supplementary Fig. 4. Construction of highly invasive H1299 cell lines by an in vitro EMT system. a qRT–PCR analysis of EMT markers (CDH2, CLDN1, MMP9) in wt-H1299 and In-H1299 cells. The mean ± SD of three independent experiments is presented. *p* values were calculated using Student's *t test* (***$p < 0.001$). **b** Wound-healing assay. Scratch assays of wt-H1299 and In-H1299 cells were performed. After 24 h, wound closure was measured. The mean ± SD of three independent experiments is presented. *p* values were calculated using Student's *t test* (**$p < 0.01$). **c** Migration (left) and invasion (right) assays of wt-H1299 and In-H1299 cells. Cell migration and invasion assays were performed after 24 h. Migrated/invaded cells were stained with crystal violet. Scale bar, 200 µm. **d** Increase in MMP9 concentration in the cell culture media using an MMP9 ELISA kit in wt-H1299 and In-H1299 cells. The MMP9 ELISA kit was purchased from Abcam. The mean ± SD of three independent experiments is presented. *p* values were calculated using Student's *t test* (***$p < 0.001$). **e** qRT–PCR analysis of SMYD2 and SMAD3 expression levels in wt-H1299 and In-H1299 cells. The mean ± SD of three independent experiments is presented. *p* values were calculated using Student's *t test* (**$p < 0.01$, ***$p < 0.001$).

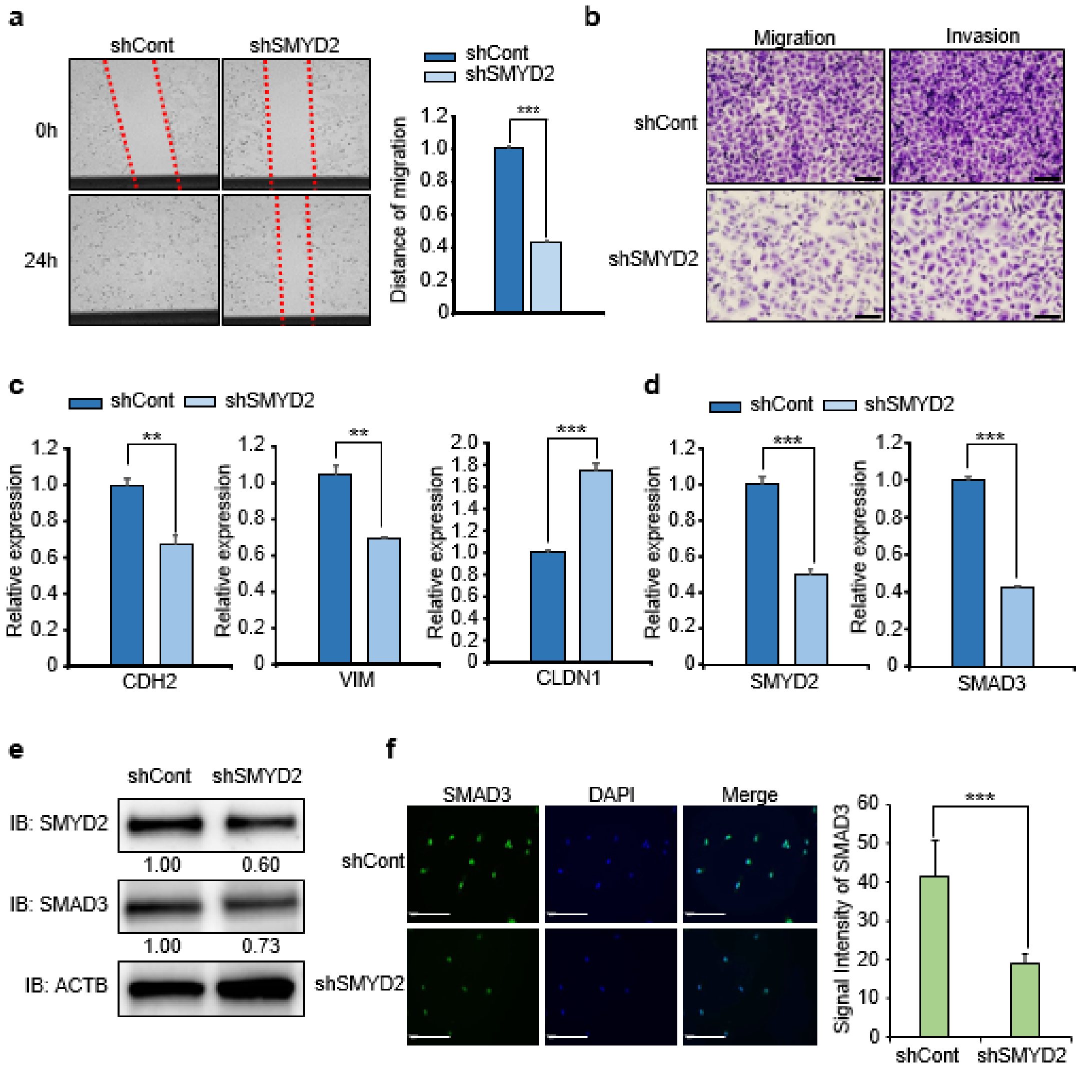

Supplementary Fig. 5. Construction of shSMYD2-H1299 cell lines. a Wound-healing assay. Scratch assays of shCont and shSMYD2 H1299 cells were performed. After 24 h, wound closure was measured. The mean ±SD of three independent experiments is shown. *p* values were calculated using Student's *t*-test (****p* < 0.001). **b** Migration (left) and invasion (right) assays of shCont and shSMYD2 H1299 cells. Cell migration and invasion assays were performed after 36 h. Migrated/invaded cells were stained with crystal violet. Scale bar, 200 µm. **c-d** qRT-PCR analysis of EMT marker expression levels (CDH2, VIM, CLDN1) (c) and SMAD3 expression levels (d) expression in shCont and shSMYD2 H1299 cells. The mean ±SD of three independent experiments is shown. *p* values were calculated using Student's *t*-test (****p* < 0.001, ***p* < 0.01). **e** Western blot analysis of SMAD3 expression levels in shCont and shSMYD2 H1299 cells. ACTB was used as the internal control. **f** Immunocytochemical analysis of SMAD3. shCont and shSMYD2 H1299 cells were fixed with 100% methanol and stained with anti-SMAD3 (Alexa Fluor 488, green) and DAPI (blue) (left). Quantification of SMAD3 expression in the immunocytochemical analysis. The mean ±SD of three independent experiments is shown. *p* values were calculated using Student's *t*-tests (****p* < 0.001) (right). Scale bar, 200 µm. The signal intensities were quantified using ImageJ software.

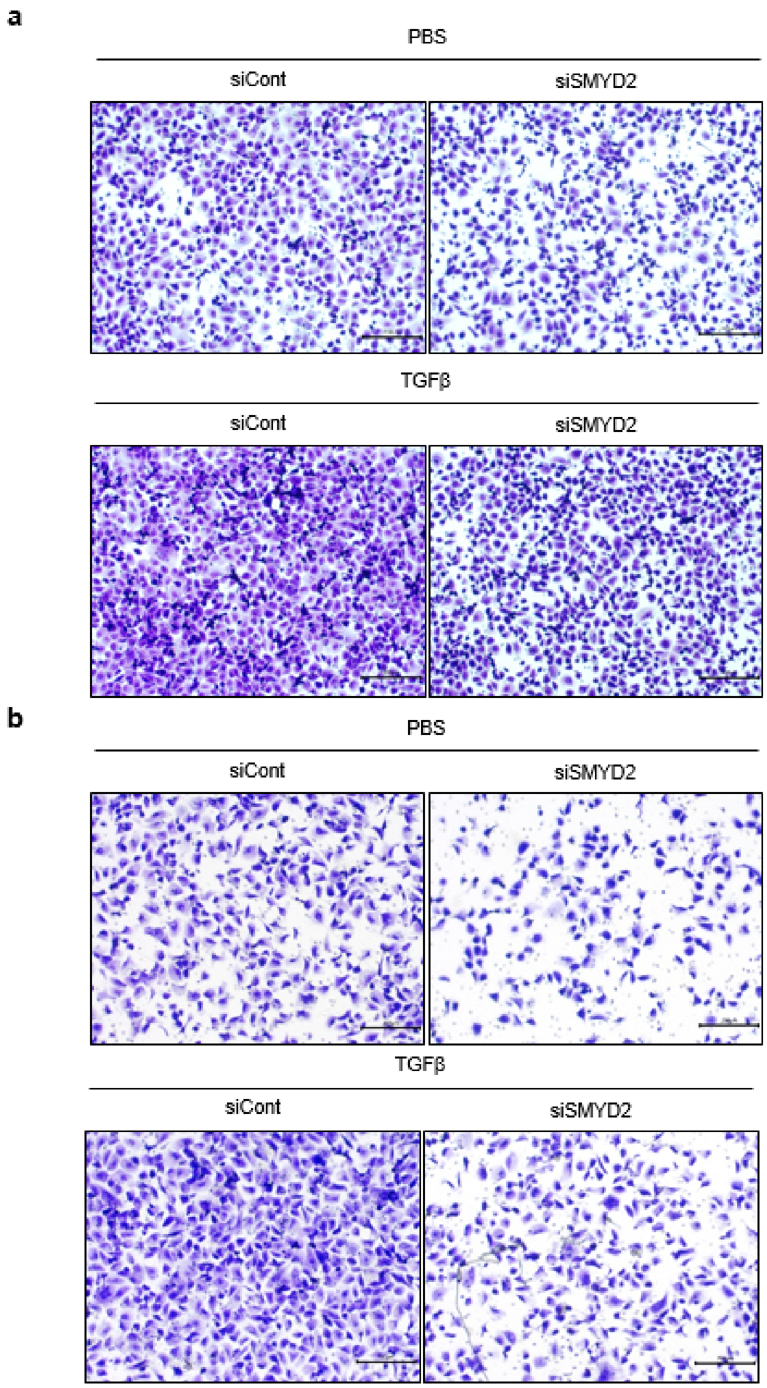

Supplementary Fig. 6. Migration and invasion are reduced by SMYD2 knockdown in TGF-β-induced EMT. a H1299 cells were treated with 10 ng/ml TGF-β followed by transfection with SMYD2 siRNA and siCont. Cell migration assays were performed after 36 h. Migrated cells were stained with crystal violet. Scale bar, 200 μm. **b** H1299 cells were treated with 10 ng/ml TGF-β followed by transfection with SMYD2 siRNA and siCont. Cell invasion assays were performed after 24 h. Invaded cells were stained with crystal violet. Scale bar, 200 μm.
